# Serological survey of *Leptospira interrogans*,* Toxoplasma gondii* and *Trypanosoma cruzi* in free roaming domestic dogs and cats from a marginated rural area of Yucatan Mexico

**DOI:** 10.1002/vms3.55

**Published:** 2017-01-11

**Authors:** Antonio Ortega‐Pacheco, Eugenia Guzmán‐Marín, Karla Y. Acosta‐Viana, Ignacio Vado‐Solís, Bertha Jiménez‐Delgadillo, Maria Cárdenas‐Marrufo, Carlos Pérez‐Osorio, Marilyn Puerto‐Solís, Matilde Jiménez‐Coello

**Affiliations:** ^1^ Campus de Ciencias Biológicas y Agropecuarias C.A. Salud Animal y Medicina Preventiva, Universidad Autónoma de Yucatán. Km 15.5 carretera Mérida‐Xmatkuil, Mérida Yucatán México; ^2^ Centro de Investigaciones Regionales‐Biomédicas “Dr Hideyo Noguchi” C.A. Biomedicina de Enfermedades Infecciosas Universidad Autónoma de Yucatán Yucatán Mexico; ^3^ Facultad de Medicina C.A. Enfermedades Endémicas Emergentes y Reemergentes en Región Tropical Universidad Autónoma de Yucatán Yucatán Mexico

**Keywords:** zoonosis, stray dogs, rural community, poverty, *Trypanosoma cruzi*, *Toxoplasma gondii*, *Leptospira* spp, free roaming pets, rural communities

## Abstract

To evaluate the serological status for *Trypanosoma cruzi*,* Toxoplasma gondii* and *Leptospira interrogans* antibodies in free roaming dogs and cats from a marginated rural community in Yucatan Mexico, 100 households were visited and animals sampled. From the 106 samples, 93 were from dogs and 13 were from cats. Frequency of positive results for *T. gondii, T. cruzi* and *Leptospira* spp was 97.8%, 9.7% and 45.2% for dogs and 92.3%, 0.0% and 15.2% for cats, respectively. No associations with age, sex and body condition was found for *T. gondii* and *Leptospira* spp neither for the place where pets sleep, fumigation or presence of triatomes in the household in the case of *T. cruzi*. For leptospirosis the most common serovars found were *Canicola, Autralis* and *Bratislava* in dogs and cats with titres of 100 or 200 with exception of one dog with a titre of 400. The high frequency of seropositive dogs suggests a high circulation of the agents in the population of free roaming owned dogs and cats probably due to the lack of control of the reservoirs and vectors involved. Domestic animals in those rural communities can be sentinels to assess the risk of human exposure in the rural communities.

## Introduction

Several infectious agents and their vectors are present widely in the domestic dogs habitats. Diseases appear when animals become in contact with the agents and their immunological response is not enough to contain them. However, overt clinical signs did not always result, especially in mild infections and dogs might become reservoirs. On the other hand, dogs may act as sentinels of several infectious agents also potentially infective in humans, so the epidemiological studies in dogs of such agents may be of value to measure the risk of infection (Salb *et al*. 2008; Schurer *et al*. 2014). Bloodsucker bug from the Triatominae subfamily transmit American Trypanosomiasis, which is endemic in Yucatan Mexico. The disease affects several hosts including dogs, cats and humans (Jiménez‐Coello *et al*. 2008, 2010). Triatomines may have feeding preferences for dogs (Guzman‐Marin *et al*. 1992; Castañera *et al*. 1998), which become important reservoirs for *T. cruzi* and are involved in an intra‐domiciliary transmission cycle (Estrada‐Franco *et al*. 2006; Gürtler *et al*. 2007). Dogs may act as important reservoirs of *T. cruzi* in the domestic and peri‐domestic cycle of transmission. A positive dogs may increase the risk of transmission to the owners and at the contrary, keeping infected dogs out of the bedroom can effectively reduce the bug and human prevalence rate (Cohen & Gürtler 2001).

Toxoplasmosis is a parasitic disease caused by the intracellular protozoan *Toxoplasma gondii* which is transmitted to humans by the ingestion of contaminated material with oocysts acquired from the environment or through the ingestion of raw meats. Cats are essentials for the biological cycle of the parasite and dogs are intermediate hosts that can also suffer from the disease and develop clinical signs such as jaundice, neurological disorders, myositis, fever, tonsillitis, dyspnoea and intraocular signs (Dubey *et al*. 2009).

Leptospirosis is considered as one of the most common zoonotic emerging infectious disease. It is produced by a spirochete bacterium of the genus *Leptospira* comprised by pathogenic and saprophytic species. According to the new classification of *Leptospira* there are at least seven pathogenic species, one of them is *L*. *interrogans* (Levett 2001). *L. interrogans* species are classified into serovars; and serovars antigenically related have been grouped into serogroups (Kmety & Dikken 1993). Currently around 200 serovars of *L. interrogans* sensu lato are recognized (Levett 2001). Those of veterinary relevance because of their zoonotic implications include serovars *Canicola*,* Grippotyphosa*,* Icterohaemorrhagiae*,* Pomona*,* Autumnalis* and *Bratislava*, originated from a specific or incidental chronically infected host. Humans become infected with leptospiras by direct contact with infected urine or indirectly by consumption of contaminated water. Thus, water‐related recreational and occupational activities pose a risk of human infection (Monahan *et al*. 2009). Dogs are common companion backyard animals in rural Mexico with proportions up to 1.7 of people/dog ratio (Ortega‐Pacheco *et al*. 2007). The close contact with animals and their feces in these systems may pose a high risk of contract zoonotic diseases like leptospirosis. In the case of cats, their role as healthy carrier as a source of contamination is likely underestimated (Hartmann *et al*. 2013).

Poor and marginalized populations are highly associated to zoonotic infections due to their low socio‐economical situation and poor hygiene, particularly endemic diseases from developing countries where the health services are inadequate (Seimenis 2012). The objectives of this study were to evaluate the serological status of free roaming owned dogs and cats for *Trypanosoma cruzi*,* Toxoplasma gondii* and *Lepstospira interrogans*, from a marginated rural community in Yucatan Mexico.

## Material and methods

### Studied area

The study was performed in a rural community (Mayapan) in the state of Yucatan, Mexico (20**°**28′ and 20**°**48′N latitude, and 89**°**12′ and 89**°**38′W longitude) with a total of 2437 inhabitants. A multistage sampling technique was designed to randomly visit 100 households from the 462 reported (INEGI 2001). Households were selected by convenience with the pre‐requisite of owning a dog and/or cat and representing at least 20% of the community. The study was performed from September to December 2015.

### Sampling

Owned free roaming dogs and cats belonging to the selected households were blood sampled from the cephalic vein or jugular vein into vacutainer tubes. Samples were centrifuged for 10 min at 400g for 15 minutes to obtain serum, were identified individually and stored at −20**°**C until processing. The owners responded a short questionnaire with the vaccination status of their dogs (including rabies and leptospirosis).

### Laboratory analysis

#### Trypanosoma cruzi

For the specific detection of IgG antibodies against *T. cruzi*, a commercial indirect enzyme‐linked immunosorbent assay (ELISA) was used (WienerLab Chagatest, V 3.0). The technique used was adapted to that described by Jiménez‐Coello *et al*. (2010, 2012), using anti‐IgG cat/dog (respectively) antibody labelled with horseradish peroxidase (HRP) on 96‐well plate coated with recombinant proteins of *T. cruzi*. Serum samples were diluted to a ratio of 100 in phosphate‐buffered saline (PBS; pH 7.2), and the secondary goat anti‐cat IgG or anti‐dog IgG HRP labelled were used (sc‐2423 and sc‐2433; Santa Cruz Inc., Santa Cruz, CA) at a dilution of 5000.

Sera from previously evaluated cats and dogs with high anti‐IgG antibody titres by ELISA (1:1024) and positive results to PCR against *T. cruzi* were used as positive controls. A sera pool from 10 healthy cats previously tested by triplicate with ELISA IgM, IgG and PCR were used as negative controls. As well commercial normal serum samples from dogs and cats were also assayed (Codes sc‐2478 and sc‐2710, Santa Cruz Inc) in each run for additional negative validated samples. For interpretation, subjects were diagnosed as either positive/negative for specific IgG antibodies to *T. cruzi* depending their optical density (OD), which were measured in a spectrophotometer at 450 nm (XMark Microplate Spectrophotometer, Bio‐Rad) and was used to compute the percent positivity (PP) using the formula mean OD (sample or negative control) divided by the mean OD value positive control multiplied by 100. Per cent positivity of 15% or above was considered as positive. For confirmation of the diagnosis only in seropositive cases previously detected by the indirect ELISA, the Western blot (WB) assay was performed (Jiménez‐Coello *et al*. 2008, 2012), in which H4 *T. cruzi* strain epimastigotes were used as antigen, where were transferred to nitrocellulose membranes. Samples were considered positives to WB based on an established criterion. A serum sample was considered positive when it recognized at least five antigenic bands from a group of 10 with the highest frequency; the result was considered indeterminate when the sample recognized of 1–4 antigenic bands and was negative when the serum sample showed no reactivity (Teixeira *et al*. 1994; Jiménez‐Coello *et al*. 2008, 2012).

#### Toxoplasma gondii

The presence of specific IgG antibodies against *T. gondii* was determined separately by the use of indirect ELISA tests (Human‐GmbH, Wiesbaden, GER), using a 96‐well plate coated with sonicated parasite proteins from tachyzoites of *T. gondii* as previously described (Castillo‐Morales *et al*. 2012). Serum samples were diluted to a ratio of 1:100 in buffer (pH 6.5 ± 0.2) provided by commercial manufacturer (phosphate‐buffer 10 mmol/L, NaCl 8 g/L and albumin 10 g/L). The secondary goat anti‐IgG dog and goat anti‐IgG anti‐IgG cat antibody HRP labelled were used (Codes sc‐2433 and sc‐2423, Santa Cruz Inc.), respectively, for each species group evaluated samples. Sera from previously assessed samples from dogs and cats showing high anti‐IgG antibodies titre by ELISA (1:1024) and positive results to PCR against *T. gondii* were used as positive controls, and sera pool from 10 healthy cats and 10 healthy cats dogs previously tested by triplicate with ELISA IgM, IgG and PCR, were used as negative controls. Also, commercial normal serum samples from dogs and cats were also assayed (Codes sc‐2478 and sc‐2710, Santa Cruz Inc) in each run. Subjects were diagnosed as either serum positive/negative for specific IgG and IgM antibodies to *T. gondii*. The optical density (OD) was measured in a spectrophotometer at 450 nm (XMark Microplate Spectrophotometer, Bio‐Rad) and was used to compute the PP using the formula mean OD (sample or negative control) divided by the mean OD value positive control multiplied by 100. Percent positivity of 15% or above was considered as positive.

#### *Leptospira* spp

To detect the presence of *Leptospira* spp antibodies, the Microscopic agglutination test (MAT) was used. MAT is the gold standard of reference for the diagnosis of leptospirosis and was performed using live antigens at the Laboratory of FM‐UADY under the WHO norms (Mayers 1985; Faine *et al*. 1999). The antigens used were from serogroups *Canicola*,* Hardjo*,* Pyrogenes*,* Panama*,* Pomona*,* Tarassovi*,* Icterohaemorrhagiae*,* Gryppotyphosa*,* Wolffi, Autumnalis*,* Australis* and *Brastislava*, all commonly associated in previous studies with illnesses in humans and animals in Yucatan (Zavala‐Velazquez *et al*. 1984; Vado‐Solís *et al*. 2002). A positive result was considered when the sera showed at least 50% agglutination with one or more serogroups, which were further, titrated in serial twofold dilutions 1:100. In cases of cross‐reaction, the serogroup with higher titres at the dilution was considered as the predominant (Mayers 1985).

### Statistical analysis

Descriptive statistics were generated to determine the prevalence of specific antibodies. Risk factor such as sex, age or body condition score (BCS) were evaluated for *T. gondii and Leptospira* spp. The association of *T. cruzi* according to place where pets sleep, fumigation or the presence of the triatomines in the household was analysed using a chi‐squared test/Fisher exact test. Odd Ratio (OR) and 95% confidence intervals (CI) were also estimated. Analysis was performed using Epi‐Info software (Version 6.0; CDC Atlanta, GA).

## Results

From the 106 samples, 93 were from dogs and 13 were cats. A high frequency of *T. gondii* seropositive animals was found in both dogs and cats. *T. cruzi* was negative in the cats, whereas in dogs, frequency was 9.7%. Leptospira spp was present in both species with a higher frequency in dogs (Table 1).

**Table 1:** Serological frequency of *T. gondii*,* T. cruzi* and *Leptospira* spp from de 93 dogs and 13 cats from a rural village of Yucatan Mexico

	Dogs			Cats		
	+	−	Frequency (%)	+	−	Frequency (%)
*T. gondii*	91	2	97.8	12	1	92.3
*T. cruzi*	8	85	9.7	0	13	0.0
*Leptospira* spp.	42	51	45.2	2	11	15.2

No associations of *T. gondii* or *T. cruzi* were found according to evaluated risk factor (Table 2).

**Table 2:** Risk factors associated with seropositivity to *Trypanosoma cruzi* in free roaming dog from a rural area of Yucatan, Mexico

Risk factor	*n*	Positive	Prevalence (%)	OR	95% CI	Chi square value	*P*‐Value
Sex							
Male	72	7	9.7	2.05	0.22–47.01		0.68 (NS)
Female	19	0 (1)	0 (1)				
Age (years)							
1<	29	2	6.9			0.11	0.95 (NS)
>1˂6	48	4	8.3				
>6	10	1	10				
BCS							
1	8	1	12.5			0.27	0.87 (NS)
2	39	3	7.7				
3–5	42	3	7.1				
Place where pets sleep							
Outdoors	83	7	7.8	0.55	0.05–13.92		0.49^ F^ (NS)
Indoors	6	0 (1)	0				
Fumigation							
Yes	70	7	10	2.1	0.23–48.53		0.67^ F^ (NS)
No	19	0 (1)	0 (1)				
Presence of the triatomes							
Yes	54	5	9.3	1.79	0.28–14.20		0.69 ^F^(NS)
No	37	2	5.4				
Place where triatomes were seen							
Indoors	37	2	5.4	0.27	0.03–2.29		0.31^ F^ (NS)
Outdoors	17	3	17.6				

OR, Odds ratio; NS, Non significant; CI, Confidence interval; F, Fisher Exact test.

The percentage of seropositivity from the 93 tested dogs was 45.2% (*n* = 42) and associated to serogroups *Canicola*,* Australis* and *Bratislava*. Only three cats from the 13 (23.2%) were seropositive to serovars *Canicola* and *Australis*. None of the studied dogs had history of recent vaccination against *Leptospira* spp.

Titres of positive animals including both species were predominantly at the dilution of 1/100 (Table 3). No association of *Leptospira* spp was found according to sex, age and BCS (Table 4).

**Table 3:** Titres of seropositive free roaming dogs and cats from a rural area of Yucatan Mexico using MAT according to the serovars of *Leptospira* spp

Titres	*Canicola*	*Australis*	*Bratislava*	*Total*
1/100	13	14	4	31
1/200	7	4	2	13
1/300	0	0	0	0
1/400	1	0	0	1
Total	21 (46.6%)	18 (40.0%)	6 (13.4%)	45

MAT, Microscopic agglutination test.

**Table 4:** Risk factors associated with seropositivity to *Leptospira* spp in free roaming dog from a rural area of Yucatan, Mexico

Risk factor	*n*	Positive	Prevalence (%)	OR	95% CI	Chi square value	*P*‐Value
Sex							
Male	72	31	43.06				
Female	18	10	55.56	0.60	0.19–1.92		0.34
Age (years)							
1<	29	12	41.38				
>1–6	47	20	42.55				
6>	10	4	40		—	0.03	0.98
BCS							
1	8	3	37.5				
2	39	19	48.72				
3.5	39	18	46.15		—	0.34	0.84

OR, odds ratio; CI, Confidence interval; NS, non significant.

## Discussion

### Toxoplasma gondii

Almost all cats were seropositive to toxoplasmosis as seen in previous studies from the same region where 91.8% of cats living in an urban area were seropositive to *T. gondii* IgG (Castillo‐Morales *et al*. 2012). Cats are definitive hosts of the protozoa and infection is expected when consuming raw meat especially in rural areas where small intermediate host vertebrates can be ready available to prey. As consequence of the uncontrolled cat population queen can reproduce all year‐round and kittens are available during the whole year (Ortega‐Pacheco *et al*. 2012) and thus a high environmental contamination with infective oocysts is expected. Other domestic animals and humans living in the same area are at high risk to become infected from drinking contaminated water with oocysts or infected meat with *T. gondii* cysts. The high frequency of *T. gondii* seropositive dogs demonstrates the wide distribution of the parasite in the environment of the studied region where cats may have experienced periods of oocyte elimination. Although a higher frequency is expected in older dogs (Langoni *et al*. 2014) a high environmental contamination with infective oocysts is possible and thus all animals in every age category are at the same risk to become in to contact with the oocysts and seroconvert.

### Trypanosoma cruzi

As seen in other rural regions of Yucatan, seropositive dogs to trypanosomiasis is expected with frequencies up to 9.8% (Jiménez‐Coello *et al*. 2008), similarly as found in this study. Dogs may be a preferred blood source for the vector (Triatomine bugs) (Guzman‐Marin *et al*. 1992; Mota *et al*. 2007), and maintain parasitaemia for longer periods of time (Gürtler *et al*. 1997). The presence of infected dogs may represent an important risk for owners when humans become infected when bitten by a vector infected by a positive dog (Jiménez‐Coello *et al*. 2010). Cats can also be a source of food for Triatoma bugs but seroprevalences in this species are seldom reported. Trypanosomiasis in cats has been reported in some areas of Argentina where together with dogs they are used as sentinels to assess the risk of human trypanosomiasis (Cardinal *et al*. 2007; Gürtler *et al*. 2007). In Yucatan a recent study including 220 domestic cats a prevalence of ELISA IgG antibodies was 8.6% and 34% of the cases amplified the sequence of kADN of *T. cruzi* (Jiménez‐Coello *et al*. 2012). Domestic cats and dogs are major domestic reservoirs of *T. cruzi* with increasing risk of peridomiciliary infection in vectors and humans. In this study, no cats were positive probably due to the low number of cats sampled. In rural Yucatan, cats are not as common as dogs and indeed the population is very reduced.

### *Leptospira* spp.

Results from this study demonstrate a wide distribution of dogs with antibodies to *Leptospira* spp. The persistence of the spirochetes in the studied region and seroprevalence in dogs may be high (up to 35%) as consequence of the environmental conditions, especially during the raining season when a high humidity may persist for months (Jiménez‐Coello *et al*. 2008). Serovars found in dogs varies from countries and regions depending on the presence of different reservoir species. For instance, in Brazil several serovars (*Canicola*,* Copenhageni* and *Pyrogenes*) are reported in dogs (Castro *et al*. 2011), whereas in the United States prevalence of antibodies in dogs was highest the serovars *Grippotyphosa*, followed by *Bratislava*,* Canicola*,* Icterohaemorrhagiae* and *Pomona* (Stokes *et al*. 2007). The serovar *Canicola* is reported in dogs from Merida together with serovar *Icterohaeomorrhagiae*, followed by *Panama* and *Pyrogenes* (Jiménez‐Coello *et al*. 2008). Similarly, as found in this study, serovar *Canicola* was more frequently found but followed by *Australis* and *Bratislava*. Serovar *Canicola* is frequently found in dogs since they are the principal reservoir host. Serovar *Australis* is common in rats and marsupials from tropical Australia, whereas Bratislava is common in pigs. Pigs, rats, skunk and opossum are common species in rural Yucatan and cross transmissions between these species and dogs may occur. This high seropositivity may suggest a wide distribution of the agent affecting both dogs and humans from the studied region, as dogs may have persistence lesptospiruria increasing the risk of transmission to other dogs and humans (Cárdenas‐Marrufo *et al*. 2011). The presence of seropositive cats, despite the low number sampled indicates the high circulation of the serovars and close contact between domestic and wild species. Cats can also be a source of contamination since they may have periods of urinary shedding (Rodriguez *et al*. 2014). This appears to be the first report of a serological survey of leptospiral infection in cats in the Yucatan area and probably in Mexico. Titres against *Leptospira* spp were within 100–200 in both species indicating exposure to the spirochete. During an active phase of the disease titres can be as high as 3200 in cats without clinical or clinic‐pathological major changes (Larsson *et al*. 1985) or >1800 in non‐vaccinated dogs (Tangeman & Littman 2013).

## Conclusions

The high number of seropositive animals with the different agents found in the population of free roaming owned dogs and cats is an evidence of past exposure and suggesting their high circulation in the studied region. Although no detected, fatal cases in either species may occur, dogs and cats may be used as sentinels for the infectious agents to assess the risk of human infection in the rural communities where control of the vectors/reservoirs is non‐existent. Surveillance in animals and people and good detection methods should be implemented by public health services to reduce the risk of human infection.

## Source of funding

This study was supported by the PRODEP (Programa de Mejoramiento de Profesores) through the academic net “Modelo de studio para el diagnóstico y prevencion de enfermedades infecciosas y parasitarias en una comunidad con bajo indice de desarrollo humano en el Estado de Yucatán (CIRB‐2012‐0008)”.

## Conflict of Interest

The author declares that they have no conflict of interest.

## Contributions

AOP participated in the design of the study, carried out the blood collection from the dogs and cats, analysis of the results and article witting. EGM, KYAV, MPS and MJC participated in the design of the study and collection of information from households, performed serological evaluation of American Trypanosomiasis, and toxoplasmosis and article writing. IVS, BJD, MCM, CPO participated in the design of the study, perform serological evaluation against Leptospira spp and article writing.
